# Automated diffusion-based parcellation of the hypothalamus reveals subunit-specific associations with obesity

**DOI:** 10.1038/s41598-020-79289-9

**Published:** 2020-12-17

**Authors:** Melanie Spindler, Jale Özyurt, Christiane M. Thiel

**Affiliations:** 1 Biological Psychology, Department of Psychology, School of Medicine and Health Sciences, Carl von Ossietzky Universität Oldenburg, Oldenburg, Germany; 2 Cluster of Excellence “Hearing4all”, Carl von Ossietzky Universität Oldenburg, Oldenburg, Germany; 3 Research Centre Neurosensory Science, Carl von Ossietzky Universität Oldenburg, Oldenburg, Germany

**Keywords:** Neuroscience, Psychology, Anatomy, Diseases

## Abstract

The hypothalamus is a small, yet highly versatile structure mainly involved in bodily functions such as control of food intake and endocrine activity. Functional anatomy of different hypothalamic areas is mainly investigated using structural MRI, validated by ex-vivo histological studies. Based on diffusion-weighted imaging (DWI), recent automated clustering methods provide robust tools for parcellation. Using data of 100 healthy adults provided by the Human Connectome Project Database, we applied DWI-based automated clustering to the hypothalamus and related microstructural properties in these hypothalamic compartments to obesity. Our results suggest that the hypothalamus can be reliably partitioned into four clusters in each hemisphere using diffusion-based parcellation. These correspond to an anterior–superior, anterior-inferior, intermediate, and posterior cluster. Obesity was predicted by mean diffusivity of the anterior–superior cluster, suggesting altered inhibition of food intake. The proposed method provides an automated hypothalamic parcellation technique based on DWI data to explore anatomy and function of hypothalamic subunits in vivo in humans.

## Introduction

The hypothalamus plays a central role in controlling bodily functions, such as endocrine activity, food intake, and energy homeostasis^1,2^ and hypothalamic abnormalities have been linked to behavioral dysfunctions in these domains^3^. For example, research suggests that functional connections between the insula and hypothalamus are related to obesity^1,4^, indicating compromised mechanisms of food intake control. Additionally, mean diffusivity (MD), reflecting overall diffusion within the hypothalamus has been associated with obesity, suggesting altered microstructural integrity^5^. Although the hypothalamus is a small structure of about 1–4 cm^3^, it is made up of approximately 15 distinct nuclei^6^ with different connections to widespread cortical and subcortical areas^7^. Nevertheless, correlates of hypothalamic function in humans have often not been related to different compartments. To better determine hypothalamic contributions to obesity and other functions, it is however of substantial interest to consider not only the hypothalamus but also its subunits.

Several methods have been proposed for hypothalamic compartmentalization in humans. Prior studies using structural magnetic resonance imaging (MRI) employed a combination of manual and semi-automated segmentation techniques based on ex-vivo histological knowledge^8,9^. Current approaches make use of anatomical landmarks visible in T1- and T2-weighted MRIs, whereby commonly identified subunits are the preoptic, anterior, tuberal and posterior/mammillary areas^8,10,11^. Still, there is a missing consensus across studies on the location and separation of hypothalamic compartments. Some authors combined different regions into one cluster (e.g. preoptic and anterior, or anterior and tuberal subunits), or used inferior to superior^9,12,13^, or ventral to lateral^7^ boundaries to define the subunits. Most approaches result in four or five compartments depending on the method used, but three or six compartments have been reported as well. To establish more standardized procedures, two very recent studies proposed the usage of automated parcellation techniques. In Billot et al.^14^, a convolutional neural network was trained on a set of manually parcellated landmark-based subregions for automated parcellation with T1w images. In contrast, a study by Neudorfer et al.^15^ proposed a detailed hypothalamic atlas containing nuclei and surrounding structures based on data from the Human Connectome Project (HCP). A summary of hypothalamus parcellation studies is given in Table 1. Still, it is unclear whether manual or automated landmark-based parcellation also reflects functional relevance. Additionally, the hypothalamus is a highly variable structure with considerable interindividual differences in size and shape that could be overlooked employing atlas-based techniques. Therefore, data-driven automated procedures could be used to capture variations in healthy and diseased populations by integrating knowledge about the underlying tissue. During the past years, advances in MR imaging enabled the development of methods for automated parcellations using local diffusion properties.

**Table 1:** Overview of studies including parcellation of the hypothalamus in humans using MRI and histology.

	Sample description	Parcellation modality	Procedure	No. subunits	Subunits (nuclei)
Baroncini et al.^8^	HC (n = 20), ex-vivo (n = 6)	1.5T T1w, T2w, histology	Manual	4	Preoptic (PN, SDN, Pe), Anterior (PVN, SO, SCh, AN, LHA), Tuberal (VMN, DMN, Inf, PeF, LHA), Posterior (MM, LM, TM, PHA, LHAp, LTN)
Billot et al.^14^	HC and frontotemporal dementia (n = 37) described in^12^, HCP (n = 2), IXI (n = 2) and ADNI dataset (n = 675)	3T T1w	Automated	5	Anterior–superior (PVN) Anterior–inferior (SO) Superior–tuberal (DMN, LHA, PVN), Inferior–tuberal (Inf, VMN, SO), Posterior (MM, LM, LHA) as in ^12^
Bocchetta et al.^12^	Frontotemporal dementia (n = 18), HC (n = 18)	3T T1w, T2w	Manual	5	Anterior–superior (PVN) Anterior–inferior (SO) Superior–tuberal (DMN, LHA, PVN), Inferior–tuberal (Inf, VMN, SO), Posterior (MM, LM, LHA)
Florent et al.^49^	Anorexia nervosa (n = 10), normal-weight (n = 10) and constitutionally lean HC (n = 10)	3T T1w	Manual	–	mammillary region, PHA, LHA a/p, VMN, DMN, SO, Inf, PVN, medial PN, tuberal LHA
Goldstein et al.^10^	Schizophrenia cohorts (n = 88), relatives (n = 45), and HC (n = 48)	1.5T T1w	Manual	4	Preoptic, Anterior, Tuberal Posterior (MM, LM)
Lemaire et al.^7^	Neurodegenerative disease (n = 7), HC (n = 7)	3T T1w, T2w	Manual	6	Preoptic, Supraoptic, Anteroventral, Anterodorsal, Lateral, Posterior
Makris et al. ^9^	Ex-vivo (n = 2), HC (n = 44)	7T T1w, 1.5T T1w, histology	Manual	5	Anterior–superior (PN, PVN, SDN), Anterior–inferior (SCh, SO), Superior tuberal (PVN, DMN, LHA), Inferior tuberal (SO, Inf, VMN, LTN), Posterior (LM, MM, LHA, TM)
Neudorfer et al.^15^	HCP dataset (n = 900) for atlas generation, hypothalamic lesion (n = 1), deep brain stimulation patients (n = 2)	3T T1w, T2w	Manual, automated (atlas)	–	AN, Inf, (dorsal) Pe, DMN, LHA, medial PN, PVN, PHA, SCh, SO, TM, VMN
Osada et al.^50^	HC (n = 12)	3T rs-fMRI	Automated	–	Inf, AN, MM,VMN, PN, DMN, PVN, PHA, LHAa/p
Piguet et al.^3^	Frontotemporal dementia (n = 18, ex-vivo: n = 12), HC (n = 16, ex-vivo: n = 6)	3T T1w, histology	Manual	2	Anterior, Posterior
Schindler et al.^11^	HC (n = 10)	7T T1w	Manual	4	Preoptic, Anterior, Tuberal, Posterior
Schönknecht et al.^16^	HC (n = 10)	3T DWI	Automated	3	Anterior (PVN, AN, DMN, LHA), Posteromedial (SCh, Inf, VMN, PHA, MM,LM), Lateral (VMN, SO, LHA)
Wolff et al.^13^	HC (n = 4), depression (n = 8)	3T T1w	Semi-automated	4	Preoptic, Intermediate–superior, Intermediate–inferior, Posterior

When parcellation was performed on nucleus level, the number of subunits is denoted as –.

*HC* Healthy controls, *PN* preoptic nucleus, *SDN* sexually dimorphic nucleus, *Pe* periventricular nucleus, *PVN* paraventricular nucleus, *SO* supraoptic nucleus, *SCh* suprachiasmatic nucleus, *AN* anterior nucleus, *LHA a/p* lateral hypothalamic area anterior/posterior, *VMN* ventromedial nucleus, *DMN* dorsomedial nucleus, *Inf* infundibular/arcuate nucleus, *PeF* perifornical nucleus, *MM* medial mammillary nucleus, *LM* lateral mammillary nucleus, *TM* tuberomammillary nucleus, *PHA* posterior hypothalamic area, *LTN* lateral tuberal nucleus.

A first approach to automated hypothalamic parcellation based on diffusion-weighted imaging (DWI) was introduced by Schönknecht et al.^16^. The authors applied k-means clustering on the principal diffusion direction information in each voxel of a manually segmented hypothalamus mask. With this approach they could reliably divide the hypothalamus into three distinct clusters according to their main diffusion direction: anterior, posteromedial, and lateral. The principal diffusion direction displays, however, only one aspect of diffusion within each voxel and does not grasp the full information of the diffusion tensor. Further, the measure is affected by crossing fibres, which are present in form of e.g., fornical fibres passing through the hypothalamus, or by the presence of cerebrospinal fluid (CSF) in voxels with close spatial proximity to the third ventricle. Therefore, the principal diffusion direction in diffusion-based clustering has recently been widely replaced by approaches utilizing more encompassing parameters to describe the diffusion process. For example, Battistella et al.^17^ employed a weighted k-means clustering algorithm on diffusion orientation distribution functions (ODF) in each voxel of an automatically created mask of the thalamus to identify anatomically meaningful subregions that were in accordance with a standard anatomic atlas of the thalamus^17,18^. Through the use of ODFs, the probability of diffusion in any direction, crossing or kissing fibres can be better characterized^19^.

The goal of the present study was twofold. First, we aimed to investigate whether the k-means clustering algorithm on diffusion ODFs can reliably compartmentalize the hypothalamus. Second, we aimed to examine the relationship between neuronal integrity in these compartments and obesity. We used a large dataset of high-resolution diffusion-weighted images from the HCP. Overall, this study provides a data-driven automated parcellation method of the hypothalamus without the use of anatomical landmarks and enhances our knowledge regarding hypothalamic anatomy and function.

## Results

Intraclass correlation (ICC) for hypothalamic volume between both raters (n = 29) was good (ICC = 0.805, 95% confidence interval 0.63–0.90). Dice coefficients ranged between 87 and 96% overlap. Body-Mass Index (BMI) of participants (n = 100) ranged between 18.44 and 41.76, with 27% of participants with a BMI ≥ 30.

### Clustering

**Figure 1:**
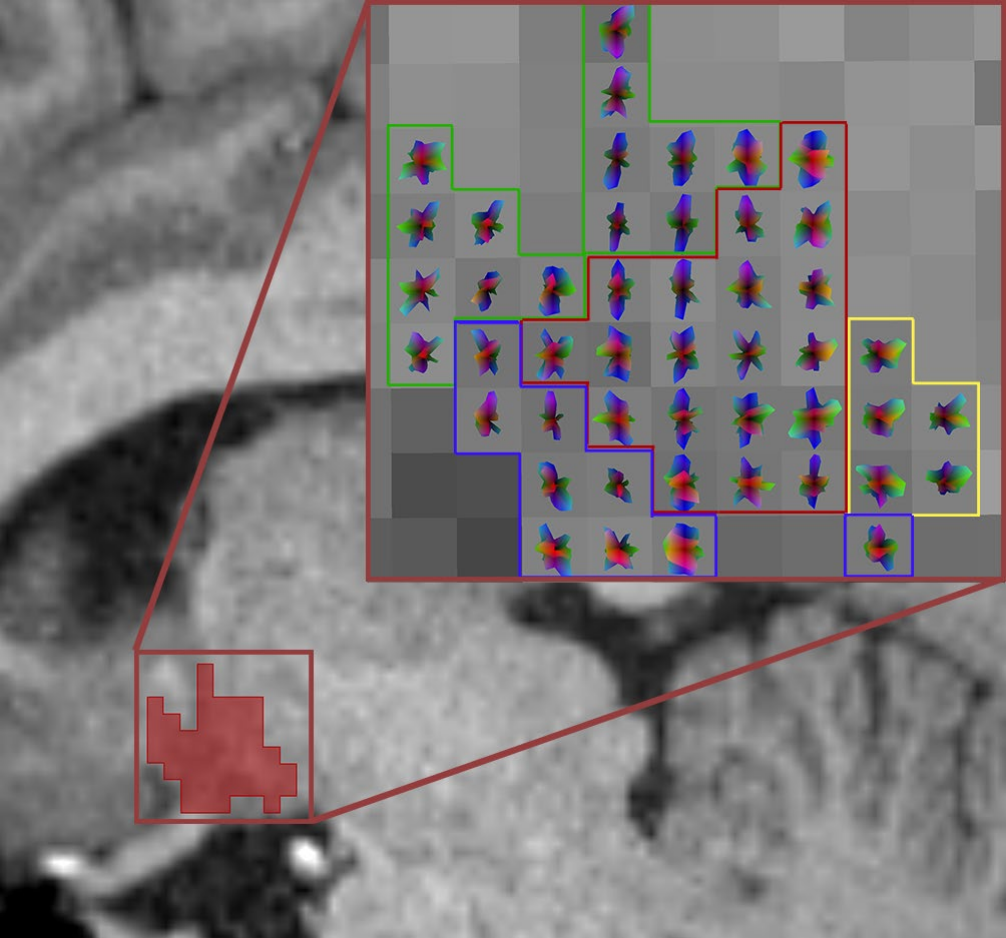
Visualization of the diffusion orientation distribution functions (ODF) and the corresponding clusters (large rectangle, anterior–superior in green, anterior-inferior in blue, intermediate in red, posterior in yellow) in a CSF- and FA-thresholded sagittal slice of the total hypothalamus (small rectangle, lateral view to display all clusters). Color-coding of the ODFs represents the probability of orientation in the given direction at each point of the surface: red for right–left, green for rostral–caudal, blue for dorsal–ventral.

**Figure 2:**
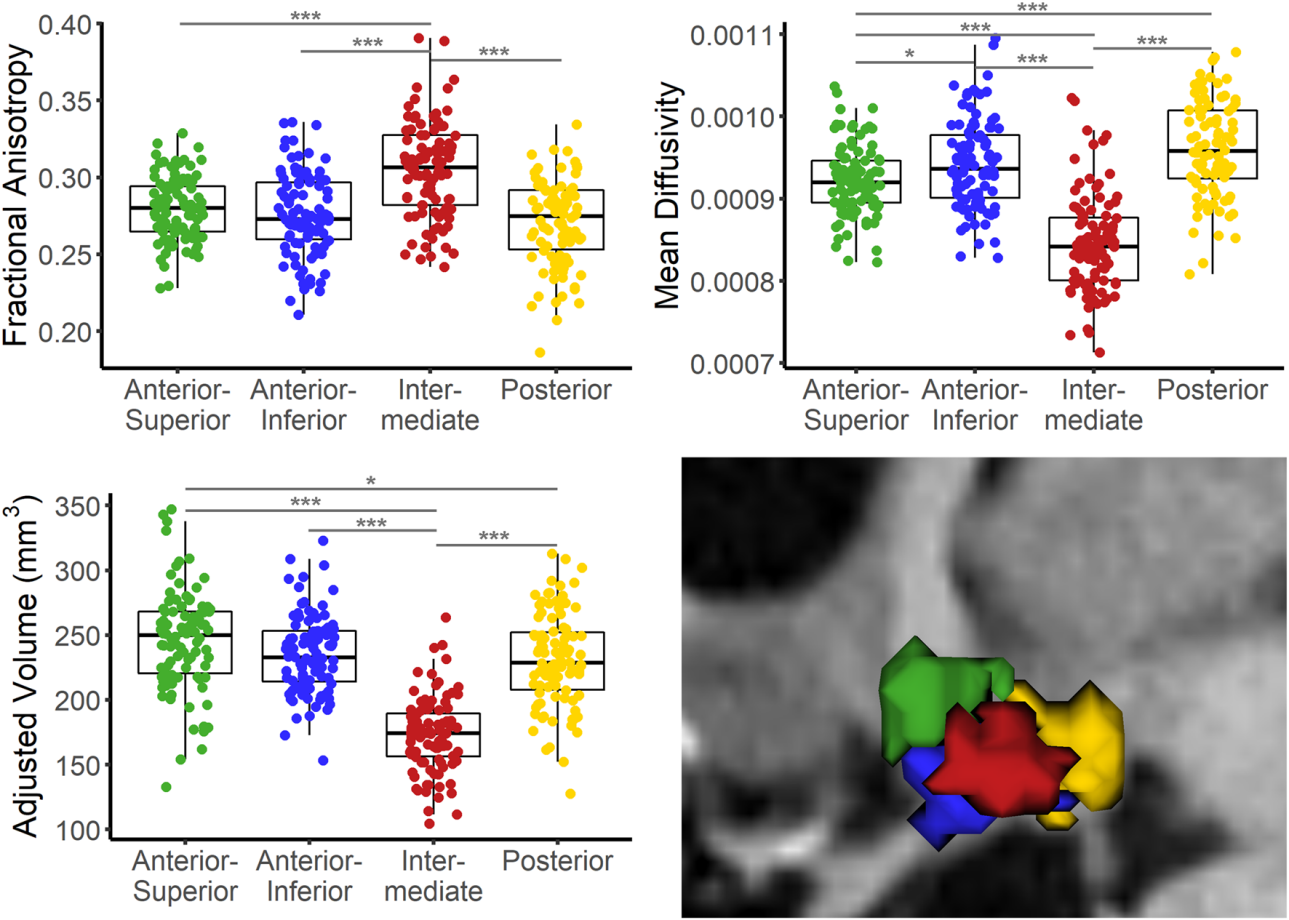
Clustering results in 3D sagittal view exemplary for one participant (bottom right), and boxplots displaying intracranial volume-corrected cluster volumes (bottom left), fractional anisotropy (top left), and mean diffusivity (top right) for each participant. Tukey’s HSD post hoc test results: ***p < 0.001, *p < 0.05.

The clustering algorithm was able to reliably identify four clusters per hemisphere, which could be divided into anterior–superior, anterior-inferior, intermediate, and posterior in 98% of the cases (Figs. 1, 2). For two participants, generated clusters could not be divided into these categories. In both cases, the anterior–inferior and intermediate clusters could not be reliably identified. Therefore, both participants were excluded from further analyses. Cluster differences in volume as well as tissue properties measured by FA and MD were assessed with three one-way ANOVAs. Results suggest that clusters differed in volume (F(3,388) = 92.83, p < 0.001), FA (F(3,388) = 32.17, p < 0.001), and MD (F(3,388) = 86.42, p < 0.001). Tukey’s HSD post hoc test results are displayed in Fig. 2.

Table 2 shows the assignment of hypothalamic nuclei to clusters obtained in the current study.

**Table 2:** Assignment of hypothalamic nuclei to clusters obtained by k-means clustering based on diffusion orientation distribution functions.

Cluster	Nuclei
Anterior–superior	Lateral and medial preoptic nuclei, paraventricular nucleus, anterior hypothalamic nucleus
Anterior–inferior	Suprachiasmatic nucleus, supraoptic nucleus, infundibular nucleus, anterior hypothalamic nucleus, ventromedial nucleus
Intermediate	Lateral hypothalamic area, dorsomedial nucleus, ventromedial nucleus
Posterior	Lateral and medial mamillary nuclei, posterior hypothalamic nucleus, tuberomammillary nucleus

To generate a probabilistic map of the hypothalamus and its subunits, individual parcellations of n = 98 subjects were registered to Montreal Neurological Institute (MNI) space and added to a common template. The resulting atlas was compared to individual parcellations of the validation sample (n = 20). In all subjects of the validation sample, subunits based on ODFs were computed and registered to MNI space. Dice coefficients between the data-driven parcellations and the atlas-based subunits suggest a high mean overlap for the anterior–superior (0.87 ± 0.05), anterior-inferior (0.78 ± 0.07), and posterior (0.83 ± 0.05) subunits, as well as medium overlap for the intermediate subunit (0.51 ± 0.14).

### Relationship between integrity of hypothalamic subunits and obesity

To investigate the relationship between hypothalamic integrity, gauged by MD, and obesity, a multiple linear regression analysis was employed with BMI as response variable (n = 98). Predictor variables included sex, age, cardiovascular fitness, and MD in each hypothalamic subunit. To test for correlation between predictors, the variance inflation factor was computed, which is a measure for inflated variance of regression coefficients due to multicollinearity. A variance inflation factor of < 2 for all predictors suggested a low correlation between predictors. The regression analysis revealed that the BMI was significantly predicted by cardiovascular fitness and MD in the anterior–superior hypothalamic subunit, explaining 12.5% of variance in BMI (F(7,90) = 2.971, p = 0.008, Adj. R^2^ = 0.125, Table 3).

**Table 3:** Results of a multiple linear regression predicting BMI.

Predictor variables	Standardized coefficients β	Standard error (SE)	T	p
(Intercept)	− 0.06	0.15	− 0.387	0.700
Anterior–superior MD	0.29	0.11	2.646	0.009*
Anterior–inferior MD	− 0.15	0.11	− 1.374	0.173
Intermediate MD	0.01	0.10	0.049	0.961
Posterior MD	0.19	0.13	1.473	0.144
Cardiovascular fitness	− 0.29	0.10	− 2.807	0.006*
Age	− 0.02	0.10	− 0.168	0.867
Sex (male)	0.12	0.24	0.509	0.612

*MD* mean diffusivity.

*p < 0.05.

Given those results, a post hoc Pearson correlation analysis was performed to examine whether anterior–superior MD and cardiovascular fitness are correlated. Results suggested no linear relationship between anterior–superior MD and cardiovascular fitness (r = 0.06, p = 0.525).

## Discussion

In this study, we propose a data-driven automated parcellation procedure based on high resolution DWI data for the hypothalamus to identify anatomically meaningful subunits based on the diffusion process in each voxel and provide information regarding the relationship between obesity and hypothalamic microstructure.

We showed that using a weighted k-means clustering algorithm based on diffusion ODFs, we were able to reliably divide the hypothalamus into four subunits (anterior–superior, anterior–inferior, intermediate, and posterior) with different underlying tissue microstructure. Here, subunits differed mostly in MD, whereas in FA, only the intermediate subunit was significantly different from the other subunits. We assume that this is related to residual white matter of the fornix that could not be fully excluded. To date, histological data serves as gold standard for hypothalamus parcellation, thus we compared our results to studies with parcellations based on histology. For example, Baroncini et al.^8^ also divided the hypothalamus into four subunits, but nuclei of the anterior–superior and anterior–inferior subunits were instead grouped from ventral to dorsal into preoptic and anterior subunits. Therefore, their subunits contain different nuclei than those in our study. Still, the tuberal and posterior region are similar to our intermediate and posterior clusters. A study conducted by Makris et al.^9^ used manual parcellation of the hypothalamus based on ex-vivo MRI and subsequent histological analysis and divided the hypothalamus into five subunits: Anterior–superior, anterior–inferior, superior–tuberal, inferior–tuberal, and posterior (Table 1). These clusters closely resemble the subunits we obtained with our automatic parcellation technique. When comparing the spatial extent of the clusters, it is, however, important to note that in our case the infundibular nucleus is more likely to be located in the anterior–inferior instead of the inferior–tuberal cluster as suggested by the authors. Due to the missing consensus concerning subunit boundaries across studies, subunits are only comparable to a limited extent. Additionally, methodological differences in rating criteria between studies add further variability to the results. Possible interindividual differences, such as displacement, shrinkage, or enlargement of nuclei may also confound the data, especially in clinical samples. Therefore, an automated procedure incorporating knowledge about underlying tissue presents a useful alternative to landmark- or atlas-based parcellations when it comes to comparing hypothalamic differences across healthy and diseased populations.

We also investigated the role of obesity in hypothalamic microstructure measured by MD. MD is commonly referred to as a measure of white matter integrity. For example, higher MD is an indicator for increased tissue water, which could be induced by e.g., inflammation or edema. Multiple neuronal mechanisms can result in changes in the diffusion tensor that in turn influence MD^20^. In obesity, hypothalamic inflammation is commonly associated with a high-fat diet, with most evidence based on animal model studies^21,22^. In obese humans, hypothalamic inflammatory markers including increased gliosis and higher mean diffusivity have been observed^5,21^. For example, Thomas et al.^5^ showed a positive association between MD in the hypothalamus and BMI. We were able to replicate the finding of increased hypothalamic MD in obesity, and notably, in our study, this increase was observed in the anterior–superior region of the hypothalamus only. Albeit a partial correlation between BMI and cardiovascular fitness was observed in our regression model, anterior–superior MD and cardiovascular fitness were not significantly correlated. Hence, it can be assumed that the relationship between hypothalamic microstructure and BMI is at least partly independent of physical activity. The anterior–superior subunit contains the paraventricular nucleus (PVN), which is suggested to play a critical role in inhibitory control of food intake and energy expenditure^23,24^. In the melanocortin system, proopiomelanocortin (POMC)-expressing neurons in the arcuate nucleus project to the PVN, where regulatory neuropeptides including oxytocin and corticotropin-releasing hormone (CRH) are secreted, which regulate feeding behaviour^25,26^. Increased MD in the paraventricular nucleus could therefore be an indicator of altered inhibitory control of food intake. Additionally, the PVN regulates hypothalamic–hypophyseal–adrenal axis (HPA axis) activity^27^, thereby presenting a link between the stress and feeding systems. Dysregulation of the HPA axis is commonly associated with obesity, explained by interactions between glucocorticoids and neuropeptides that mediate feeding behavior^28^. For example, activation of the PVN stimulates the release of adrenocorticotropic hormone, which contribute to an increased production and release of glucocorticoids that in turn alter the expression of neuropeptides (e.g., POMC)^29^. Therefore, future research should consider both stress-related and metabolic mechanisms when exploring neural correlates of obesity. Also likely influenced by obesity and related to hypothalamic microstructure is the hypothalamic–pituitary–gonadal (HPG) axis^30^. Here, it is suggested that energy balance and adipose tissue affect the reproductive system via Gonadotropin-releasing hormone that is produced in the hypothalamus^31^. But even in healthy individuals, the hypothalamus is affected by activity of the HPG axis. For example in 2010, Baroncini et al. found changes in hypothalamic MD during the course of the artificial menstrual cycle^32^. Due to the multifunctional role of the hypothalamus, other mechanisms influencing our results cannot be ruled out, and as the generated clusters in this study contain multiple nuclei of the hypothalamus, interpretations on the basis of single nuclei remain speculative. Additionally, MD is a rather unspecific, albeit sensitive DTI measure^20^. It should also be noted, that due to the proximity of the hypothalamus to the third ventricle, especially the periventricular and arcuate nucleus are most likely not fully included in our masks. Nevertheless, our results add to previous findings indicating that obesity is associated with changes in specific subunits of the hypothalamus^33^, highlighting the importance of hypothalamic parcellation when investigating hypothalamic function.

We conclude that automated parcellation methods based on DWI could be a useful tool for assessing hypothalamic microstructure. We further provide evidence of a relation between hypothalamic microstructure, particularly in the anterior–superior region, and obesity. The multifaceted role of the hypothalamus in endocrine, cognitive and metabolic functions highlights the importance of studying unique features of hypothalamic subunits. Additionally, automated parcellation procedures constitute a useful addition to unravelling hypothalamic function in health and disease.

## Methods

### Participants

One hundred unrelated healthy subjects (age range 22–36, 54 females) provided by the HCP database were used. As validation sample, twenty additional subjects (age range 23–35, 8 females) were randomly selected from the HCP database. All subjects were scanned on a customized 3T Connectome Scanner adapted from a Siemens Skyra (Siemens AG, Erlangen, Germany) with a 32-channel head coil and completed behavioral assessment at Washington University^34,35^. In addition to basic demographic information (age in years, sex), measures for obesity and cardiovascular fitness were selected from the available data. The HCP protocol was approved by the Institutional Review Board at Washington University in St. Louis and data acquisition was in accordance with the declaration of Helsinki^36^. All participants provided written informed consent for the project.

### MRI data

T1-weighted high-resolution anatomical images were obtained using a 3D Magnetization-Prepared Rapid Acquisition Gradient Echo (MP-RAGE) sequence with the following parameters: sagittal acquisition, isotropic voxel size of 0.7 mm^3^, echo time (TE) = 2.14 ms, repetition time (TR) = 2400 ms, echo spacing (ES) = 7.6 ms, inversion time (IT) = 1000 ms, flip angle (FA) = 8°, field of view (FOV) = 180 × 224 × 224 mm, and bandwidth (BW) = 210 Hz per pixel. The acquisition time was 7 min 40 s. T2-weighted sagittal images were acquired using a variable flip angle turbo spin-echo sequence (Siemens SPACE^37^) with an acquisition time of 8 min 24 s, the same voxel size, FOV, and slices as in the T1w, BW = 744 Hz per pixel, TE = 565 ms, TR = 3200 ms, and ES = 3.53 ms. Diffusion-weighted images were acquired using a single-shot 2D spin-echo multiband sequence with multiband factor 3, and the following parameters: TR = 5520 ms, TE = 89.5 ms, ES = 0.78 ms, FA = 78°, voxel size = 1.25 mm^3^, b-values 1000, 2000 and 3000 s/mm^2^, BW = 1488 Hz per pixel, and FOV = 210 × 180 × 138 mm. Three different gradient tables were used, each acquired once with left-to-right and right-to-left phase encoding direction. Each of the gradient tables included approximately 90 diffusion-weighting directions plus 6 b = 0 images interspersed throughout each run. Acquisition time was around 55 min.

### MRI processing

We obtained minimally preprocessed T1-weighted, T2-weighted, and DWI data from the HCP database (S1200 release)^35,38^, where T1-weighted images were aligned to anterior–posterior commissure (AC-PC) orientation in the midsagittal plane, and DWIs were corrected for susceptibility field distortions, eddy currents and subject movement using the FMRIB Software library tools^39^ (FSL, https://fsl.fmrib.ox.ac.uk/fsl), and registered to the T1-weighted image. A downsampled T1-weighted image matching the DWIs was generated as well. In addition, we performed segmentation of T1 images into grey matter (GM), white matter (WM) and CSF, with bias-correction using the Statistical Parametric Mapping toolbox (SPM12, https://www.fil.ion.ucl.ac.uk/spm/software/spm12/), running on MATLAB 2017a (Mathworks Inc.). From the preprocessed DWI data, we extracted the b_1000_ shell and fitted a tensor model to generate Fractional Anisotropy (FA) and MD maps for each participant.

### Segmentation of the hypothalamus

#### Hypothalamus extraction

To generate a mask of the left and right hypothalamus, we used a semi-automated approach. First, the Segment Editor in Slicer v.10.4.2^40^ in triplanar view (https://www.slicer.org/) was used for manual slice-by-slice segmentation on coronal slices. The high resolution T1-weighted image was superimposed onto the downsampled (1.25 mm^3^) T1 image such that anatomical structures were better visible during segmentation. Axial and sagittal views were used to allow easier identification of landmarks (e.g., the mammillary bodies, thalamus). The segmentation procedure started on the most anterior coronal slice where the anterior commissure appeared continuous and ended on the last slice with visible mammillary bodies^11^. Parts of the fornix surrounded by hypothalamic tissue were included for all participants, as they could not be reliably excluded. The superior fornix was excluded. The left and right hemispheric masks were created separately for each participant. Afterwards, the masks were checked using the T2-weighted image, to accurately delineate especially the lateral boundary of the hypothalamus. Segmentation took approximately 30 min per participant. For a more detailed description of the segmentation procedure, see^10,11,13^. To control for partial volume effects, the hypothalamus masks were then automatically refined using CSF probability maps calculated in SPM12, whereby voxels with a probability of > 15% CSF were excluded from the mask. To further exclude voxels containing the optic nerves or the fornix, an FA threshold of > 0.55 was employed as well. Both thresholds were based on previous research^18^ and empirically adjusted to minimize the loss of hypothalamic structures close to the ventricles. Consistency of manual hypothalamus segmentation was determined by analysis of inter-rater reliability with a second independent rater using a data subsample (n = 29). Inter-rater reliability was calculated with intraclass correlation (model 3.1)^41^ and Dice coefficients^42^. Masks generated by researcher 1 were used for final computations across the whole sample.

#### Clustering of the hypothalamus

To reconstruct the fibre orientations in the hypothalamus, we estimated the ODFs within the hypothalamus using the qboot command implemented in FSL (Fig. 1). Qboot uses constant solid angle q-Ball imaging to generate ODFs based on spherical harmonics (SH). The maximum SH basis was set to 6 (lmax = 6), whereas the number of ODF peaks was kept at 2, with 50 samples of residual bootstrapping (default settings). Single-shell data with b = 1000 was used to obtain results comparable to the most widely used DWI sequences.

Clustering was applied using a procedure adapted from Battistella et al.^17^. In short, the approach combines the Euclidean distances of voxel position coordinates and ODF coefficients with an equal weighting of 0.5 as input into a k-means clustering algorithm. Hereby, the spatial distance served to constrain the clusters to be contiguous, while the distance between the ODF coefficients grouped voxels with similar microstructural diffusion properties. First, 5000 randomly initialized k-means were run with only voxel position as input to generate the average centroid which served as the initial setting for the final clustering. To eliminate the influence of voxel size on clustering with the position coordinates and to achieve an easier adaptation across different imaging sequences, we introduced a standardization of both ODF coefficients and voxel coordinates to have M = 0 and SD = 1. This approach served to obtain a similar range of Euclidean distances and replaced multiplication of the ODFs by a constant as proposed in the original paper^17^.

The optimal number of clusters was evaluated between k = [2, 3, 4, 5, 6] as observed in the literature, by minimizing the Davies-Bouldin index^43^ in 20 randomly selected participants. The Davies–Bouldin index measures similarity as the ratio of within-cluster to between-cluster distances and is a commonly used method to determine a proper number of clusters. Here, the optimal number of clusters ranged between 2 and 6, with k = 2: 2.5%, 3: 10%, 4: 32.5%, 5: 25%, and 6: 30% of the cases. Afterwards, the spatial position of clusters obtained with k = 4, 5, and 6 clusters was assessed, and it was determined that in contrast to k = 5 or 6, for k = 4, the same spatial formations could be identified in all participants. Depending on their location, clusters were labelled as anterior–superior, anterior–inferior, intermediate, or posterior. Volumes for each cluster were corrected for total intracranial volume (ICV) using the residual approach^44,45^. Mean volume, FA, and MD were then compared between clusters using three one-way ANOVAs (p < 0.017 considered significant after Bonferroni adjustment).

To determine cluster overlap between subjects, hypothalamic subunits were affine and non-linearly registered to the MNI template in 1mm^3^ resolution using FSL’s flirt and fnirt (n = 98). Spatial overlap of clusters was resolved such that a voxel was assigned to the cluster with the highest probability (majority voting). Voxel values then represented the number of participants that share the cluster label at the respective voxel, with higher values at the center and lower values at the borders of each cluster. The resulting atlas template was made publicly available (https://github.com/SpindM/HypothalamicAtlas), and can be further thresholded to only include voxels exceeding a certain probability for more conservative or liberal masks. To determine individual correspondence of atlas-generated subunits with those of individual data-driven parcellation, further n = 20 participants from the HCP database were included as a validation sample and analyzed following the procedures described above. Afterwards, subunits were registered to MNI space, and Dice coefficients were calculated between atlas-based and data-driven subunits.

### Relationship between integrity of hypothalamic subunits and obesity

We included information about body-mass index (BMI) measured in kg/m^2^ and scores of a 2-min walk test measuring sub-maximal cardiovascular fitness. The test was adapted from the American Thoracic Society’s 6-min Walk Test Protocol^46^, and recorded the distance that the participant was able to walk on a 50-foot (out and back) course in 2 min. The raw score was calculated as the distance (in feet and inches) walked. For analyses, we used age-adjusted scale scores. Including instructions and practice, the test took about 4 min. To investigate the relationship of hypothalamic microstructure with the Body Mass Index, a multiple linear regression analysis was computed to predict BMI from mean diffusivity in each hypothalamic cluster, controlling for sex, age in years, and cardiovascular fitness. To detect possible collinearity among predictors, the variance inflation factor was computed. A Pearson correlation was conducted exploratively to analyze the association between anterior–superior MD and cardiovascular fitness. If not stated otherwise, statistical significance was set at p < 0.05. Behavioral analyses were conducted using R (v. 3.6.3)^47^. Visualizations were performed with FiberNavigator^48^ and Slicer^40^.

## Data Availability

The data used for this study was accessed from https://db.humanconnectome.org and is publicly available. The group template of hypothalamic subunits in standard MNI space is publicly available at https://github.com/SpindM/HypothalamicAtlas.
